# Gitelman syndrome: a rare life‐threatening case of hypokalemic paralysis mimicking Guillain–Barré syndrome during pregnancy and review of the literature

**DOI:** 10.1002/ccr3.1122

**Published:** 2017-08-17

**Authors:** Abdelghafour Elkoundi, Noureddine Kartite, Mustapha Bensghir, Nawfal Doghmi, Salim Jaafar Lalaoui

**Affiliations:** ^1^ Department of Anesthesiology and Intensive Care Military Hospital Mohammed V Faculty of Medicine and Pharmacy of Rabat Mohammed V University Rabat Morocco

**Keywords:** Controversial drugs, Gitelman syndrome, Guillain–Barré syndrome, hypokalemic paralysis, outcome, pregnancy

## Abstract

In rare cases, patients with Gitelman syndrome may present with hypokalemic paralysis mimicking Guillain–Barré syndrome. The severity of resultant symptoms may be life‐threatening. Controversial drugs such as aldactone, amiloride, and eplerenone should be used in this situation despite the lack of safety data.

## Introduction

Gitelman syndrome (GS) is a rare renal disorder resulting from a mutation in the gene SCL12A3, which encodes for the thiazide‐sensitive sodium chloride cotransporter in the distal convoluted tubule 1. It is characterized by hypokalemia, hypomagnesemia, hypocalciuria, metabolic alkalosis, and normal blood pressure. Many symptoms of this rare renal disorder are related to these electrolyte abnormalities, and adversely affect the patient's quality of life. Moreover, rarely patients with GS may present with hypokalemic paralysis mimicking Guillain–Barré syndrome (GBS) and cause diagnostic and therapeutic delay.

The present case report illustrates particularly the difficulties in the management of hypokalemic paralysis during pregnancy and the impressive complications encountered in a patient with GS. This article also focuses on the differential diagnosis of other similar electrolyte‐induced clinical disorders and the investigations required to distinguish between these conditions.

## Case Presentation

A 22‐year‐old woman (height 160 cm; weight 50 kg; BMI 19.5 kg/m^2^), gravida 1 para 0, at 16 weeks of gestation (WG) presented to the emergency department complaining of lack of ability to walk and severe weakness of the lower limbs. She had no history of symptoms similar to GS, and there was no family history of neuromuscular diseases. She previously had been in excellent health apart from mild hyperemesis gravidarum, which resolved spontaneously at approximately 10 WG without medication. She had no diarrhea and denied taking herbal medicine, diuretics, laxatives, or glucocorticoids prior to her illness.

The patient was in her usual state of health when 5 days back she noticed progressive fatigue and bilateral muscle cramping predominating in lower extremities. The patient noticed knee buckling with frequent falls. Rapidly, within that span, the muscle weakness worsened, and she was confined to bed and unable to mobilize her legs, when she decided to consult in the hospital.

Her vital signs on examination were heart rate 130/min, blood pressure 100/65 mmHg, respiratory rate 22/min, oxygen saturation 99% on room air, and temperature 37°C. The patient's review of systems was found negative for shortness of breath, cough, and swallowing difficulties. On neurological examination, the patient was alert and well‐oriented. Her motor strength in upper limbs was 4/5. Hip flexors, quadriceps, and hamstrings were 1/5 bilaterally; dorsiflexors and plantar flexors were 0/5 bilaterally. Her cranial nerve examination was intact, and the sensation was diminished to touch and pinprick on lower extremities. Cranial and neck muscles were normal, but examination revealed deep tendon reflex to be absent on knees and ankles. The examination of other systems was unremarkable.

Table 1 shows the initial laboratory data of the patient. Serum level of potassium was 2.2 mmol/L, serum magnesium 0.57 mmol/L, creatinine 68 *μ*mol/L, and urea 2.8 mmol/L. Serum levels of sodium, total calcium, glucose, cortisol, thyroid, and liver function tests were found to be in normal range. Other blood investigations showed the following: hemoglobin 12.4 g/dL, white cell count 7.5 × 10^3^/*μ*L with a neutrophil count 5.9 × 10^3^/*μ*L, and C‐reactive protein 5 mg/dL. Serum creatine phosphokinase level was 55 UI/L.

**Table 1:** Results of the laboratory investigations

Test	Values	Normal range
Complete blood count		
WBC (×10^3^/*μ*L)	7.5	4–10
Hemoglobin (g/dL)	12.4	12–16
Platelets (×10^3^/*μ*L)	180	150–450
Hematocrit (%)	37.2	40–52
Serum chemistry (mmol/L)		
Sodium	137	135–145
Potassium	2.2	3.6–5.4
Magnesium	0.57	0.7–1.1
Calcium	91	90–100
Phosphore	1	0.80–1.45
Chloride	105	101–111
Glucose	4.44	
Other investigations		
Urea (mmol/L)	2.8	2.5–7
Creatinine (*μ*mol/L)	68	50–110
Creatinine kinase (IU/L)	55	0–195
Plasma renin activity (ng/mL/h)	2.6	0.12–1.59
Aldosterone (ng/dL)	720	80–365
CRP (mg/dL)	5	<6
Procalcitonin (ng/mL)	0.1	<0.5
TSH (mIU/L)	2.12	0.34–5.60
Serum cortisol (*μ*g/dL)	21.34	8.70–22.40
Serum osmolality (mOsm/kg)	280	275–295
Urine chemistry		
Potassium (mmol/L)	51.5	16–83
Calcium (mg/L)	22	66–200
Chloride (mmol/L)	91	73–167
Creatinine (mmol/L)	11.5	3.5–23
Arterial blood gas		
pH	7.48	7.35–7.45
PCO_2_ (mmHg)	43.5	35–45
HCO_3_ (mEq/L)	37.3	22–31
PO_2_ (mmHg)	90	80–105
O_2_ saturation (%)	99	90–100
Cerebrospinal fluid analysis		
White cells count (/mm^3^)	0	<3
Protein (g/L)	0.25	0.18–0.45
Glucose (mmol/L)	2.8	2.5–3.5

WBC, white blood cell; TSH, thyroid‐stimulating hormone.

Her electrocardiogram (EKG) exhibited sinus tachycardia with inversion of T wave in leads D III, V1 to V4 suggesting hypokalemia.

She was assessed by a neurologist, and a diagnosis of Guillain–Barré Syndrome (GBS) was suspected. Her time nerve conduction studies (Table 2) revealed increased distal motor and sensitive latencies and reduced conduction velocities predominating in low members suggestive of acute demyelinating polyradiculoneuropathy, in keeping with GBS. Her cerebrospinal fluid (CSF) analysis revealed normal white cell count, protein, and glucose levels. The infectious and autoimmune screen was nonrevealing, and a fetal ultrasound scan showed an ongoing pregnancy at the age of 16 weeks and 6 days.

**Table 2:** Nerve conduction studies

Nerve	Velocity	Latency	Amplitude
Motor conductions			
Median (right)	50	4.1	0.5
Ulnar (right)	64	2.4	2.8
Posterior tibial (right)	22	7.8	0.3
Sensory conductions			
Median	NA	NA	NA
Ulnar	29 (right); 44 (left)	5.8 (right); 3.2 (left)	6 (right); 8 (left)
Sural (right)	25	7	2.5

NA, not available.

Latencies are in milliseconds, compound muscle action potential amplitude in millivolts, sensory nerve action potential amplitude in microvolts, velocities in meter per second.

**Figure 1:**
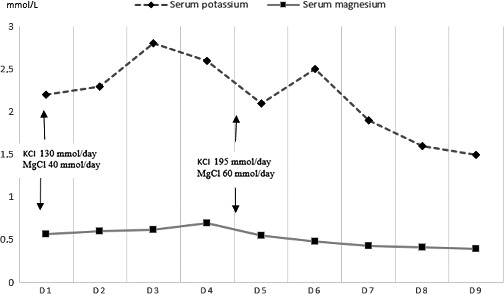
The patient's potassium and magnesium levels during hospital stay.

She was then shifted to ICU, and the next day, a course of IV immunoglobulins (0.4 g/kg per day for 5 days) was started, but this therapy resulted in a very poor effect. She was also given a continuous potassium supplementation in form of potassium chloride infusion (130 mmol/day) and intravenous magnesium chloride (40 mmol/day) under cardiac monitoring. Her muscle weakness worsened over 5 days of the treatment and spread to the upper limbs, trunk, and neck muscles. There was no associated difficulty in breathing or swallowing at this time. Serial biochemical evaluations during her hospital stay revealed persistent hypokalemia and hypomagnesemia despite parenteral supplements (Fig. 1). This was further investigated by urine analysis, which showed the potassium to be 51.5 mmol/L (normal range 16–83), chloride: 91 mmol/L (normal range 73–167), calcium: 29 mg/L (normal range 66–200 mg/L), and creatinine: 11.5 mmol/L (normal range 3.5–23). Arterial blood gas analysis was performed with the patient breathing room air, which showed metabolic alkalosis (pH 7.48, HCO_3_ 37.3, PaCO_2_ 43.5). She also had increased plasma renin activity (2.6 ng/mL/h; normal range 0.12–1.59) and serum plasma aldosterone (720 ng/dL; normal range 80–365). Under the presenting circumstances, a tubulopathy causing renal potassium loss was considered. Based on the findings of hypokalemic metabolic alkalosis without hypertension, severe hypomagnesemia, and hypocalciuria, a diagnosis of GS was suspected. This diagnosis was ascertained with the sequencing of the implicated gene, which confirmed GS as a heterozygous mutation in SLC12A3.

It was decided to reinforce ion supplementation, so potassium and magnesium supplements were increased to respectively 195 and 60 mmol/day.

On day 7 of her hospitalization, her condition deteriorated, and her tidal volume and minute volume decreased with swallowing difficulty. Oxygen saturation was 90% at 10 L/min delivered via a mask. To prevent hypoxemia and avoid emergency intubation, she was intubated and ventilated on controlled mandatory ventilation mode, and her vital parameters (electrocardiogram, invasive blood pressure, oxygen saturation, and temperature) were monitored. At this point, hypokalemia of 1.9 mmol/L was evident along with magnesium 0.43 mmol/L. Periodic fetal evaluation documented fetal well‐being and normal size fetus with normal liquor and Doppler.

On day 8, the hemodynamic status worsened and the patient experienced prolonged hypotension and intermittent episodes of bradycardia, which occurred spontaneously, without relation to vagal maneuvers, such as endotracheal suctioning. She required vigorous volume replacement with normal saline and the use of vasopressor (Norepinephrine infusion) to maintain her mean arterial blood pressure up to 65 mmHg. EKG examination showed sinus bradycardia with the rate of 45 and prolonged QT interval. Echocardiographic and Doppler assessment failed to reveal any abnormalities in myocardial morphology or function.

On day 9, spontaneously fetal demise was observed, and the patient developed ventricular fibrillation (VF). She underwent electrical cardioversion (200 J biphasic), which restored sinus rhythm. Serum level of potassium was 1.5 mmol/L, and serum magnesium was 0.4 mmol/L. In view of the recent clinical deterioration, the use of a potassium‐sparing diuretic or aldosterone antagonist was discussed, but this approach could result in hypovolemia and possibly worsen the hemodynamic status. Spironolactone was finally administered to this patient (100 mg/day). Subsequently, she continued to present repeated VF on the same day, which was complicated by a refractory cardiac arrest despite advanced cardiac life support.

## Discussion

Guillain–Barré syndrome is an inflammatory peripheral neuropathy presenting as bilateral, symmetric, ascending weakness frequently involving bulbar, and respiratory muscles. In this case, the rapid progression of the proximal motor weakness, together with areflexia of which these are the two cardinal symptoms led to an initial diagnosis of GBS 2. The patient also showed other features, which strongly support the diagnosis of GBS, including relative symmetry of symptoms, and the absence of fever at the onset. Electrophysiological testing demonstrated characteristic findings of demyelination in keeping with the diagnosis. However, there were certain contrasting features that raised doubt about this diagnosis. History of cough, fever, diarrhea, sore throat, or any other types of infections, which precede the onset of weakness in the majority of GBS patients, was not observed in this case. Unlike this case, sensory involvement and increased sensitive latencies are not usually seen in typical cases of GBS. Also, CSF examination routinely shows raised proteins with a normal white cell count. This albuminocytological dissociation was not found in the patient, as one would expect to find in GBS.

Dyselectrolytemia is also uncommon in GBS 3, 4, 5, 6, 7, and the presence of hypokalemia in such patients calls for consideration of another diagnosis.

In this case, the diagnosis was approached sequentially. Hypokalemia can result from poor potassium intake, increased translocation into the cells, or increased losses in the urine or the gastrointestinal tract. The patient was questioned regarding diarrhea, use of laxatives or insulin, excessive bicarbonate supplements, and episodic weakness. There were no arguments regarding those elements. In addition, the patient did not consume alcohol and was on a regular diet. Measurement of urinary spot potassium (mmol/L)/creatinine (mmol/L) (K/C ratio), as well as blood pressure and assessment of acid–base balance, was helpful. A spot urine K/C ratio <1.5 suggests poor intake, shift into the cells or gastrointestinal loss 8, and if hypokalemia is associated with paralysis, hyperthyroidism, barium poisoning, and familial or sporadic periodic paralysis should also be considered. However, this patient did not fit into those conditions because the K/C ratio in her case was >1.5. In addition, there was no history suggestive of thyrotoxicosis, barium poisoning, or similar episodes of weakness. The paralytic attacks in hypokalemic periodic paralysis are characterized by reversible flaccid paralysis but typically sparing the respiratory muscles and heart, unlike our patient.

The use of blood pressure status and acid–base was of great help in the differential diagnosis. In a patient with a urine K/C ratio of 1.5 or higher, two categories based upon the presence or absence of hypertension should be considered: In the presence of hypertension, renovascular hypertension and mineralocorticoid excess should be considered. As the patient was normotensive at admission, all those causative factors were ruled out. In the absence of hypertension, coexistent metabolic acidosis is consistent with renal tubular acidosis, while coexistent metabolic alkalosis is observed in prolonged vomiting, diuretic abuse, Bartter syndrome (BS), or GS. This patient denied taking diuretics, and differential diagnosis with chronic vomiting was made through the normal value of urine chloride. Hyperemesis gravidarum encountered at 10 WG in the patient was mild, and this alone is unlikely to be the primary cause of such profound hypokalemia.

Bartter syndrome and its variant GS are hypokalemic disorders caused by renal tubular defects. In BS, the site of defect is the thick ascending loop of Henle, whereas in GS, the distal convoluted tubule is affected. They both share clinical findings, such as hypokalemia, metabolic alkalosis, and activation of the renin–angiotensin–aldosterone axis. Hypocalciuria, a typical feature of GS, is absent in BS. Hypomagnesemia is also a hallmark of Gitelman syndrome. In GS, there is a disruption of sodium chloride reabsorption in the distal convoluted tubule (DCT). This causes vascular volume contraction leading to an activation of the renin–angiotensin–aldosterone axis. Further, the elevated aldosterone leads to increased sodium reabsorption in the collecting tubules, which result in increased secretion of potassium and hydrogen ions, responsible of hypokalemia and metabolic alkalosis. Inhibition of sodium entry into the DCT causes membrane hyperpolarization. The calcium channels open and calcium penetrates leading to hypocalciuria. Elevated magnesium excretion in the proximal tubule is assumed to cause hypomagnesemia.

The patient fulfilled all of the diagnostic criteria for GS. She had low serum potassium and magnesium, while her urine calcium levels were low with inappropriately high urine potassium level. These features in constellation with metabolic alkalosis, normal blood pressure, and increased plasma renin and aldosterone activity are congruous with GS. Pregnancy potentially exacerbated the electrolyte losses seen in the patient due to dilution, increased fetal demands 9, and hyperemesis gravidarum 10. In addition, renal blood flow and glomerular filtration rate are increased. To maintain electrolyte balance, there is a compensatory increase in tubular reabsorption, but in GS, this process is impaired. The diagnosis was ascertained with the sequencing of the implicated gene, which confirmed GS as a heterozygous mutation in SLC12A3. However, there are reports of pseudo‐GS, which present with GS‐like renal tubulopathy without mutations in SLC12A3 11.

This case presents particular characteristics. Firstly, the GS was not diagnosed before hospital admission, which is a rather atypical finding for the condition in pregnant patients. Profound hypokalemia with paralysis is rarely seen as the presentation in GS. Cruz et al. reported that 6% of patients with GS present with hypokalemic paralysis 12. The underlying mechanism of profound hypokalemia in this group of patients is not clear.

An extensive review of the literature revealed no other case report in which the patient had presented like GBS, but later the cause was found out to be GS. Coexistence of complex dyselectrolytemia secondary to GS in a male patient with GBS was previously described but as isolated entities 7.

There is still the question of how to explain the electrophysiological findings in the patient. During hypokalemic paralysis like in GS, muscle fibers are unexcitable, or only a small number of fibers with very low conduction might be stimulated. The derangement of serum electrolyte concentrations indeed has been shown to affect both nerve conduction velocity and the amplitude of evoked responses 13. Also, neuronal inexcitability is postulated to occur consequently upon possible inactivation of the sodium–potassium pump by the low concentration of extracellular potassium 14, 15. This hypothesis was also supported by Cruz‐Martinez et al., as they reported inexcitable muscle fibers during an acute hypokalemic attack, along with the associated slowing of conduction velocity along muscle fibers in situ 16. Similar clinical–electrophysiological discrepancy is also observed in Guillain–Barré syndrome. This could explain that when the potassium depletion is severe, as happened in the present case, the patient may develop a flaccid paralysis that may be mistaken for Guillain–Barre syndrome 17. Alternatively, potassium depletion may also be associated with necrotizing myopathy 18. Atrophic muscle fibers could contribute toward reducing conduction velocity along muscle fibers. However, muscle fiber atrophy should not be the main cause of conduction slowing, as myopathy can be absent.

Involvement of sensory nerves in hypokalemic paralysis is suggested to arise through dorsal root ganglia having an incomplete blood–nerve barrier 19, and sensory neurons being particularly vulnerable to hypokalemia.

Although the outcome is generally considered to be good, the present case report illustrates the impressive complications encountered in a pregnant patient with GS. Respiratory failure and the requirement for mechanical ventilation were observed in this patient as consequence of the profound dyselectrolytemia and respiratory muscle weakness. Such a presentation is rarely observed 20. Her intensive care unit stay was also complicated by life‐threatening episodes of hemodynamic instability related to autonomic dysfunction. The patient presented a VF cardiac arrest. The possible triggering factors include the background of profound hypokalemia and hypomagnesemia that can lengthen the duration of cardiomyocyte action potential, hence prolonging the QT interval, and thereby led to increased ventricular arrhythmogenicity. Hormonal effects on the heart and hemodynamic changes as seen in pregnancy along with increased metabolic requests may also add to their role in causing severe cardiac complications. This can explain the fact that she presented with the GS symptoms for the first time in pregnancy. In addition, the abnormal regulation of vascular tone known in GS patients may cause limited myocardial perfusion during increased metabolic requests such as pregnancy 21. Also, increased production of nitric oxide and increased angiotensin 1–7 levels expose to arrhythmias and sudden cardiac death in these patients 22. Correspondingly, Riviera‐Munoz et al. described a small subgroup of patients with a remarkable severe phenotype, including an early onset, severe neuromuscular manifestations, and ventricular arrhythmias 23. The presence of this phenotype can probably explain the similarity and the critical outcome in this patient.

The occurrence of serious cardiac arrhythmias in this patient with severe hypokalemia and hypomagnesemia may be triggered by acute events. Concomitant gastrointestinal troubles can favor it 21. However, in this case, neither diarrhea nor vomiting was observed. Septic shock is also an established trigger of cardiac events 24, 25. The results of the septic screen were unremarkable: C‐reactive protein of 7 mg/L, white cells count of 4.7 × 10^9^/L, and neutrophils of 3.2 × 10^9^/L. Procalcitonin was 0.1 ng/mL, and blood and urine cultures demonstrated no bacterial growth.

The possible triggering factor, in this case, is an enhanced response of pacemaker current to *β*‐adrenergic stimulation. It can be hypothesized that the sinoatrial node pacemaker cells, highly sensitized by massive *β*1‐adrenergic catecholamine stimulation due to treatment related, resulted in a high heart rate output and contributed substantially to the occurrence of malignant arrhythmia. In addition, norepinephrine may have caused an additionally decrease in plasma potassium, probably by shifting potassium into the cell and then predisposed to cardiac arrhythmias 26.

Fetal demise was observed in this case. In the literature, there has been only one case of such complication despite normalization of serum potassium reported by Lakhi 27.

This patient clearly illustrates the difficulties in the management of hypokalemic paralysis during pregnancy. The management of such patients is essentially based on potassium and magnesium supplements. It appears that the main challenge during pregnancy is maintaining normal serum potassium and magnesium levels but strict normalization of these ions levels in pregnancy is difficult to achieve in the majority of reports. No consensus recommendations exist for the management and the optimal targets for potassium and magnesium levels of these patients. The intravenous supplementation used was more aggressive comparatively to other reports of GS in pregnancy. Spironolactone 28, amiloride 29, 30, and eplerenone 31 can be used, but their use in pregnancy is controversial for safety reasons. It is recommended that their use must be weighed against the risk of not taking them. After fetal demise and given the expressed need to improve the maternal condition, the use of these agents seems to be an opportunity but we were aware of the risk of hypovolemia and a possible worsening of the hemodynamic status. A posteriori, and given the poor outcome, it is deemed that the use of these drugs should have been considered very early in our therapeutic approach. Therefore, an earlier use of controversial drugs when the patient presents with paralytic attack is highly suggested.

## Conclusion

The occurrence of severe hypokalemia in patients with classic characteristics of GBS at the time of presentation can cause a diagnostic and therapeutic dilemma. The constellation of hypokalemia, hypomagnesemia, hypocalciuria along with metabolic alkalosis, normal blood pressure and increased plasma renin and aldosterone activity is characteristic of GS. Although the syndrome is often mild, the severity of symptoms may be dramatic and result in life‐threatening consequences. Through this clinical case, an earlier use of controversial drugs is suggested when the patient presents with paralytic attack in order to prevent respiratory failure and malignant arrhythmias.

## Authorship

AE: drafted the manuscript and collected data. All authors: critically revised the manuscript and gave their final approval of the version to be published.

## Consent

Written consent for publication was obtained from the family of the patient described in this manuscript.

## Conflicts of Interest

The authors report no declarations of interest.
